# Portable Analyzer for On-Site Determination of Dissolved Organic Carbon—Development and Field Testing

**DOI:** 10.3390/ijerph15112335

**Published:** 2018-10-23

**Authors:** Heinrich Glorian, Viktor Schmalz, Paweł Lochyński, Paul Fremdling, Hilmar Börnick, Eckhard Worch, Thomas Dittmar

**Affiliations:** 1 Institute of Water Chemistry, Technische Universität Dresden, 01069 Dresden, Germany; viktor.schmalz@tu-dresden.de (V.S.); paul.fremdling@chem.ox.ac.uk (P.F.); hilmar.boernick@tu-dresden.de (H.B.); eckhard.worch@tu-dresden.de (E.W.); thomas.dittmar@tu-dresden.de (T.D.); 2 Institute of Environmental Engineering, Wrocław University of Environmental and Life Sciences, 50-375 Wrocław, Poland; pawel.lochynski@gmail.com

**Keywords:** DOC, on-site analysis, electro-oxidation, BDD, hydroxyl radicals, in-situ carrier gas

## Abstract

Dissolved organic carbon (DOC) is a sum parameter that is frequently used in water analytics. Highly resolved and accurate DOC data are necessary, for instance, for water quality monitoring and for the evaluation of the efficiency of treatment processes. The conventional DOC determination methods consist of on-site sampling and subsequent analysis in a stationary device in a laboratory. However, especially in regions where no or only poorly equipped laboratories are available, this method bears the risk of getting erroneous results. For this reason, the objective of the present study was to set up a reliable and portable DOC analyzer for on-site analysis. The presented DOC system is equipped with an electrolysis-based decomposition cell with boron-doped diamond electrodes (BDD) that oxidizes the organic compounds to carbon dioxide. Within this study, the influence of different electrode materials and the composition of the applied electrolytes on the DOC decomposition in an undivided electrolytic cell were systematically investigated. Furthermore, some technical aspects of the portable prototype are discussed. After a detailed validation, the prototype was used in an ongoing monitoring program in Northern India. The limit of detection is 0.1 mg L^−1^ C with a relative standard deviation of 2.3% in a linear range up to 1000 mg L C^−1^. The key features of the portable DOC analyzer are: No need for ultra-pure gases, catalysts or burning technology, an analyzing time per sample below 5 min, and a reliable on-site DOC determination.

## 1. Introduction

Dissolved organic carbon (DOC) is an indispensable analytical parameter for water quality control. Since DOC is a parameter with extensive information value, this applies to both drinking and wastewater analysis as well as to the monitoring of aquatic ecosystems. For the assessment of the quality of water bodies or the evaluation of the efficiency of drinking and wastewater treatment processes, DOC holds important information since organic contaminations can be detected safely within a very short time [1]. Using an on-site DOC analyzer, it is even possible to monitor water treatment plants with a focus on precursors, which leads to a formation of disinfection byproducts [2,3]. At present, almost exclusively stationary laboratory devices are used for this purpose. These devices are essentially based on two different decomposition processes, namely wet chemical oxidation and high-temperature combustion [3]. Alternative methods for the determination of organic compounds in water are based on the principle of UV absorption [4] or on atomic spectrometric techniques [5]. The need for availability of high-purity oxidizing agents, catalysts, high temperatures, and carrier gases hinders a mobile application of conventional systems. Novel approaches, such as the application of high-resolution spectroscopic absorption measurements, still have insufficient detection limits and are matrix-dependent to a large degree [4]. The availability of suitable on-site analysis techniques is particularly important in countries with insufficient laboratory infrastructure, since the storage and transport of samples may lead to significant uncertainties in the measurement results [6]. Therefore, portable analyzers can considerably contribute to local- and high-resolution detection of DOC.

The outlined application gap can be overcome by a novel DOC analyzer concept based on electrochemical oxidation of the organically bound carbon [7]. The core component of this analyzer is an undivided electrolysis cell equipped with a boron-doped diamond electrode (BDD). Here, an in-situ generation of ^•^OH radicals with high current efficiency takes place at the surface of the BDD, see Equation (1). These ^•^OH radicals will be used for the electrochemical oxidation of the present organic compounds (R), as it is shown in Equation (2), in a simplified manner [8,9]. ^•^OH radicals have a very high oxidation potential (E_0_ = +2.85 V) and are able to mineralize organic compounds to a great extent [10,11,12]. The CO_2_ formed during oxidation can be determined by means of a non-dispersive infrared (NDIR) detector.

BDD[ ] + H_2_O → BDD[^•^OH] + H^+^ + e^−^ (1)

R + BDD[^•^OH] → BDD[ ] + CO_2_ + H_2_O + … (2)

Here, BDD[ ] represents free adsorption sites at the anode surface where a discharge of water can take place. Because of the continuous generation of ^•^OH radicals, the concentration close to the BDD surface is in great excess in comparison with the organic compounds. For that reason, the ^•^OH radical concentration can be included in the rate constant k_t_, obtaining a pseudo-first-order reaction rate equation [13,14,15]:

ln(c_0_/c) = −k_t_ × t (3)

Here, c_0_ is the initial concentration, and c the concentration dependent on time t. The DOC decomposition can also be evaluated regarding the applied volume-weighted electrical charge Q_V_, which describes the charge introduced per unit volume V of the electrolyte dependent on the current I and the electrolysis time t as shown in Equation (4).

Q_V_ = I × t/V (4)

As a consequence, Q_V_ is affected by an increased sample volume. Since Q_V_ is the most important process variable for the electrolytic generation of ^•^OH radicals, and consequently the DOC decomposition depends on Q_V_, it is reasonable to include this parameter into the pseudo-first-order reaction rate equation:

ln(c_0_/c) = −k_t_ × t = −k_t_ × Q_V_ × V/I = −k_Q_ × Q_V_ (5)

As described above, CO_2_ is generated as a result of the DOC decomposition. The transport of the CO_2_ to the NDIR detector can be realized by means of the electrolysis gas, also generated in-situ, as shown in Equations (6) and (7). Furthermore, the movement of the fine-bubble electrolysis gases improves the mass transport inside the electrolytic cell substantially.

BDD[^•^OH] → BDD[ ] + 1/2 O_2_ + H^+^ + e^−^ (6)

2 H_2_O + 2e^−^ → H_2_ + 2 OH^−^ (7)

The general suitability of this novel approach for practical on-site applications was demonstrated in previous studies [15]. The main objective of the present study was to construct a portable prototype with an optimized analytical procedure and technical design. In this context, a complete and fast mineralization of DOC through the selection of a convenient electrolyte and suitable electrodes was of particular concern. Finally, a robust prototype with short analysis time and a sufficient limit of quantification for the assigned tasks was intended. The resulting prototype was tested and evaluated during an environmental monitoring within an ongoing research project in India. In the course of this project, the efficiency of riverbank filtration (RBF) in Northern India is comprehensively investigated. Besides selected trace pollutants, sum parameters like DOC should be monitored as well to assess the water quality improvement during RBF.

## 2. Materials and Methods 

### 2.1. Development and Setup of a Portable Prototype

The miniaturized undivided electrolytic cell was constructed according to the optimization experiments in former studies [15]. The plane electrode surface served as a leak-proof side boundary of the electrolytic cell. For conditioning purposes, a current of 2 A was applied on the electrodes until a stable current-voltage response was reached. 

Three different cathode materials were tested: Stainless steel (Fe/Cr_18_Ni_10_, Chempur GmbH, Karlsruhe, Germany), Titan (Chempur GmbH, Karlsruhe, Germany), and BDD (Condias GmbH, Itzehoe, Germany). For the BDD anode, thin-film electrodes based on niobium and carbon substrate with a different crystallinity of the deposited diamond layer and effective surface area were tested. For this purpose, DOC decomposition experiments were conducted as described below. 

**Figure 1:**
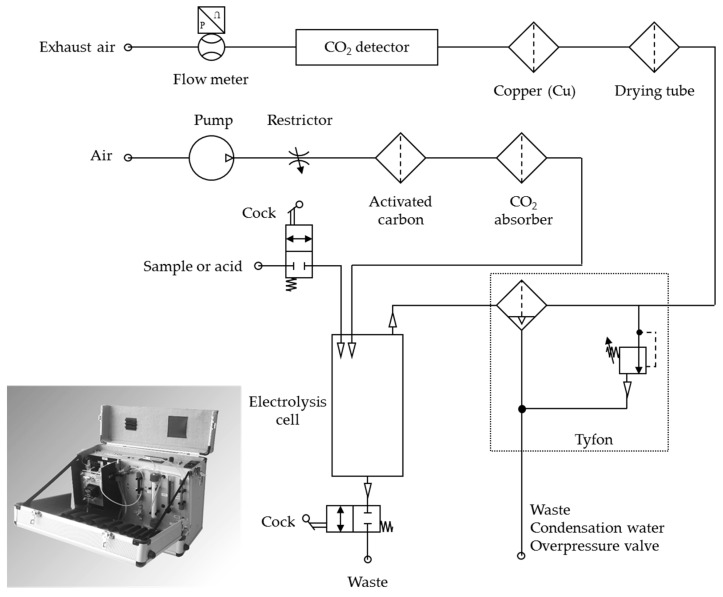
Schematic structure of the portable prototype (DIATOC).

All ports for sample inlet and outlet as well as for the gas stream were laid through the cathode. As shown in Figure 1, the gas stream was led from the cell to a moisture trap followed by a static drying tube (Me 12, Perma Pure, Lakewood, NJ, USA) and, afterward, through a glass tube filled with Mg(ClO_4_)_2_ (Eltra, Haan, Germany) to completely dry the gas stream. Following this, the gas passed a copper scrubber for the removal of elemental halogens and finally reached the detection unit. A commercial grade NDIR detector (PMM-1020-NM-P830; AidE GmbH, Dessau, Germany) was used as the detector. 

In addition to the internally generated carrier gas, external carrier gas was applied for the removal of inorganic carbon. Using a diaphragm pump, ambient air was conveyed and cleaned with activated carbon (Merck, Darmstadt, Germany) and a CO_2_ absorber (Sherasorb (NaOH, Ca(OH)_2_), Intersurgical, Sankt Augustin, Germany). The cleaned air was transferred into the electrolytic cell to purge inorganic carbon and, optionally, to support the CO_2_ transport through the system during the decomposition. The transport of CO_2_ was systematically investigated with internal as well as external carrier gas. For this purpose, an inorganic carbon standard solution made from dried sodium carbonate (Merck, Darmstadt, Germany) and sodium hydrogen carbonate (Merck, Darmstadt, Germany) was used according to the standard DIN EN 1484 H3 [16].

The DOC decomposition cell was constructed according to previous study results [15]. An electrode gap of 0.3 cm and a sample volume of 7 mL were used, which resulted in an active electrode surface of 23.33 cm^2^. The cell was run in galvanostatic mode with a current of 4 A, which led to an internal carrier gas volume of 45 mL min^−1^. The electrolytic cell was installed in a portable prototype constructed together with Elektrochemie Halle GmbH, Germany, see Figure 1. The portable prototype was set up in an aluminum suitcase with an external power connection. With a consumption of around 60 Wh, a battery power supply is also possible. 

### 2.2. DOC Decomposition Experiments

DOC stock solutions of pyridine and potassium hydrogen phthalate (KHP) with a concentration of 1000 mg L^−1^ were prepared and used for the DOC decomposition experiments (p.a., Fa. VWR, Germany). Generally, KHP was used for all experiments unless specified otherwise. From this stock solution, a diluted standard was prepared daily. For dilution, the electrolytes described below were used. A sample volume of 7 mL was filled in the electrolytic cell and flushed with 90 mL min^−1^ external carrier gas until no further inorganic carbon could be detected. Turning on the current started the decomposition. Due to the galvanostatic operation of the electrolytic cell with 4 A, 45 mL min^−1^ an internal carrier gas was produced and mixed with 45 mL min^−1^ of an external carrier gas, unless specified otherwise. As soon as the slope of the detector signal was ≥−3 μV min^−1^, the current was switched off and the measurement was completed.

### 2.3. Optimization of the Electrolyte Composition

For an accelerated decomposition of the organic compounds in the water sample, additional in-depth investigations regarding the electrolyte were carried out within extensive experimental tests. Besides investigating the influence of the type of mineral acid used for the electrolyte, the influence of sulfate ions for an advanced generation of secondary oxidants such as peroxodisulfate was intended to be observed as well. All other experimental conditions were kept as described above. All the electrolytes used are listed in Table 1. For all samples, the total amount of oxidizing agents, the hydrogen peroxide concentration, and pH were determined.

The electrolyte based on HNO_3_ had to be optimized because the electrolyte showed an insufficient stability. During the decomposition experiments using HNO_3_ as the electrolyte, an increasing cell voltage could be observed depending on the introduced charge. The rising voltage is caused by a decreasing conductivity with a simultaneous rise of the pH through a cathodic reduction of the electrolyte. Experiments with H_2_SO_4_ under the same conditions showed no significant change in cell voltage, conductivity, or pH over time and introduced charge. Due to the stable voltage, the power consumption is lower. Therefore, H_2_SO_4_ is the preferred electrolyte.

Another reason for modifying the electrolyte was the finding that it can influence the decomposition kinetics of different compounds. In previous studies, it was shown that DOC decomposition kinetics, based on ^•^OH radicals, mainly depends on the chemical structure of the respective compounds [15,17,18]. For example, the mineralization of pyridine is significantly slower than the mineralization of the commonly used DOC standard compound KHP. For accurate analysis results, a comparably fast decomposition of all constituents of DOC is desirable. If H_2_SO_4_ is used as the electrolyte, sulfate is introduced into the system. In the presence of sulfate, a generation of secondary oxidants such as peroxodisulfate is possible, in addition to a direct formation of SO4^−•^ radicals at the BDD surface [19,20,21]. The longer lifetime of these SO4^−•^ radicals compared to ^•^OH radicals could enable the transport of these oxidants from the anode surface to the bulk [22]. As a result, the DOC decomposition becomes uncoupled from the BDD surface and is significantly accelerated [23]. While the oxidation by ^•^OH radicals occurs primarily by addition to double bonds and by hydrogen abstraction, the oxidation by SO_4_^−•^ radicals is mainly based on a direct electron transfer [24]. The mechanism of the peroxodisulfate and SO_4_^−•^ radicals synthesis on BDD electrodes has been studied extensively. On the basis of density functional theory calculations, it was shown that, contrary to earlier assumptions [19,25], SO_4_^−•^ radicals are preferentially generated by a direct electron transfer at the anode [21]:

HSO_4_^−^ → SO_4_^−•^ + e^−^ + H^+^ (8)

SO4^−•^ radicals can also be formed through the previously generated ^•^OH radicals, see Equation (9) [21,25]. Peroxodisulfate is then formed by a subsequent combination of two SO4^−•^ radicals, see Equation (10) [21].

HSO_4_^−^ + ^•^OH → H_2_O + SO_4_^−•^ (9)

SO_4_^−•^ + SO_4_^−•^ → S_2_O_8_^2−^ (10)

### 2.4. Method Validation

For method validation, an electrolytic cell equipped with a stainless steel plate (Fe/Cr_18_Ni_10_, Chempur GmbH, Karlsruhe, Germany) as a cathode and a BDD thin-film electrode on a niobium substrate (DiaCCon GmbH, Fürth, Germany) as an anode were used. The prototype equipped with this cell was validated according to DIN 32645 [26] to determine the limit of detection and the limit of quantification. 

### 2.5. Field Testing

**Figure 2:**
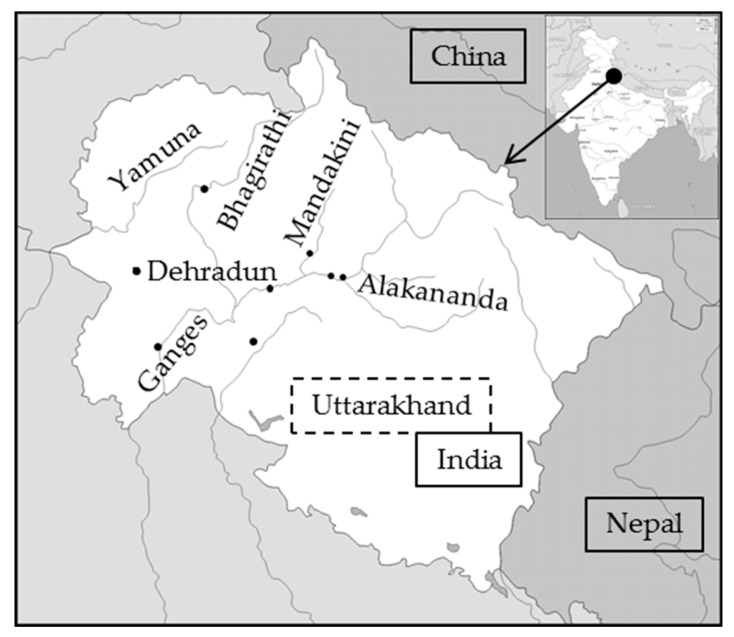
Map with sampling locations in Uttarakhand, India.

To prove the suitability of the prototype as a portable DOC analyzer, it was used during an environmental monitoring program on the water quality in Northern India. Overall, around 100 samples were taken from different sites and rivers in Uttarakhand, see Figure 2, during two sampling campaigns in October 2016 and October 2017. The water samples were taken from rivers and canals as well as from riverbank filtration and groundwater wells. All samples were filtrated (Chromafil^®^ GF/PET-45/25; Macherey-Nagel, Düren, Germany), acidified (pH 1.4, H_2_SO_4_, Merck, Darmstadt, Germany) on-site and shortly thereafter measured using the portable prototype. A comparative sample was taken, filtrated, acidified using HCl (pH 1.4, Merck, Darmstadt, Germany), refrigerated, and measured with a TOC-5000 (Shimadzu, Kyoto, Japan) as described above.

### 2.6. Chemical Analysis

#### 2.6.1. Total Oxidizing Agents

To determine the total oxidant agents formed in the solution, an iodometric method was used. It was derived from the work of Liang, Bennedsen, and Classes [27,28,29], adapted, and carefully validated. The method is based on the oxidation of I^−^ to I_2_ in NaHCO_3_ buffered medium by all relevant oxidizing agents like hydrogen peroxide or peroxodisulfate. I^−^ reacts immediately with I_2_ to form the lemon-yellow I_3_^−^.

Procedure: For calibration purposes, a 7.4 mmol L^−1^ K_2_S_2_O_8_ stock solution in ultrapure water was prepared. From this stock solution, a diluted standard solution with a concentration of 740 μmol L^−1^ was prepared. Totals of 0.5 g NaHCO_3_ and 4 g KI are weighed into a 50 mL volumetric flask. Both salts are dissolved in approximately 30 mL ultrapure water. The sample or the diluted standard solution can then be added. The resulting oxidizing agent concentration should be between 9 and 95 μmol L^−1^. Afterward, the flask is filled up with ultrapure water. The extinction is measured after 20 min at 352 nm in a 0.5 cm quartz glass cuvette. 

#### 2.6.2. Hydrogen Peroxide

The photometric H_2_O_2_ detection is based on the oxidation of potassium titanium oxalate to a yellow-orange titanium (IV) peroxide complex [30]. The detection is highly specific for H_2_O_2_ and was performed as described by the Water Reuse Foundation [31].

#### 2.6.3. Further Parameter

The determination of pH was conducted with a Sen Tix^®^ 41 pH electrode (WTW, Weilheim, Germany). Electrical conductivity was measured with an LF 323 device (WTW, Germany). In the case of comparison measurements, DOC was determined as NPOC with a TOC-5000 (Shimadzu, Japan) according to the standard DIN EN 1484 H3 [16].

## 3. Results

### 3.1. Development and Technical Optimization of the Portable Prototype

#### 3.1.1. Influence of the Degree of Crystallinity of the BDD Anode

Electrodes are the core components of the electrolysis cell. Therefore, the suitability of different anode materials was tested in order to build an optimized decomposition cell. The BDD anodes on a niobium base came from two commercial suppliers, DiaCCon GmbH and Condias GmbH, Germany. By use of the DiaCCon-BDD, a DOC decomposition rate constant of k_Q_ = 0.0364 mL A^−1^ s^−1^ could be attained. This is significantly faster than the decomposition rate obtained by using the Condias-BDD (k_Q_ = 0.0229 mL A^−1^ s^−1^). The reason was suspected to be the different crystallinity and the resulting specific surface area. To prove this assumption, DOC decomposition experiments with different BDD-SiC-C anodes were conducted. Their basis is a silicon carbide (SiC) coated graphite of defined roughness, which has been coated with boron-doped diamond using the HF-CVD (Hot Filament Chemical Vapor Deposition) method [32]. In this way, various crystallinities could be produced with particle sizes in the range of 0.1 to about 2.2 μm, which resulted in the different surface areas listed in Table 2. It was observed that anodes with a higher crystallinity showed an increased DOC decomposition. This means that the BDD surface structure crucially affects the electrochemical reactivity. Therefore, a BDD with high crystallinity should be used to reach as effective and complete a decomposition as possible [33].

#### 3.1.2. Selection of the Cathode Material

Besides the selection of an optimal anode material, a suitable cathode material had to be found. For this purpose, three different types of electrodes, stainless steel, titanium, and a BDD cathode, were tested with a focus on their influence on the DOC decomposition. Regardless of the used cathode material, no significant influence (k_Q_ variation max. 6%) on the decomposition was observed. Nevertheless, a difference in the cell voltage (U) could be found. Regardless of the electrolyte used, the difference between U_BDD_ and U_Steel_ was 1.7 V, whereas the starting cell voltage of titanium was positioned between them. Hence, out of the three tested materials, steel, with the lowest cell voltage, is the most appropriate cathode material because the power consumption of the on-site device should be as low as possible.

#### 3.1.3. Transport of the Generated CO_2_

##### Influence of External Carrier Gas

**Figure 3:**
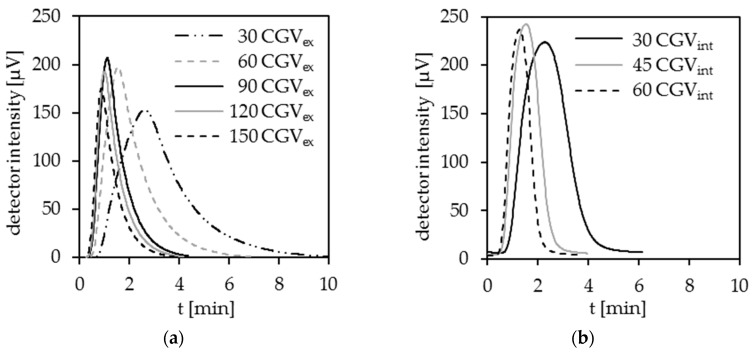
(**a**) CO_2_ transport with external carrier gas; (**b**) CO_2_ transport with internal carrier gas; HNO_3_, pH = 1.4, κ = 17 mS cm^−1^, (I = 3–6 A), stainless steel cathode, DiaCCon BDD.

For an accurate and efficient DOC analysis, a fast and lossless transport of the formed CO_2_ to the detector is of essential importance. As shown in Figure 3a and Table 3, the external carrier gas volume (CGV) has a significant influence on the signal shape. An increase of the external CGV_ex_ from 30 mL min^−1^ to 90 mL min^−1^ leads to a significantly better shape of the signal curve with a 55% reduced peak width or rather the analysis time and a decreased tailing factor by 10%. A further increased CGV_ex_ causes only a minor reduction in the analysis time, while the tailing factor rises again and the sensibility, by means of the peak area, decreases even further.

##### Influence of Internal Carrier Gas

Depending on the current, electrochemically-formed internal carrier gas is generated at the surface of the anode as well as the cathode. This gas is formed with a high reproducibility. Therefore, it can be used for the CO_2_ transport to the detector. In Figure 3b, the detector signal in the case of CO_2_ transport with the internally generated carrier gas is shown. Since the volume of the internally generated carrier gas directly depends on the applied current, it is limited by the cell voltage. According to the specification of the manufacturers, the applied BDD are nearly permanently stable up to a maximum voltage of 15 V. As shown in Table 3, an increased CGV_int_ from 30 to 45 mL min^−1^ significantly enhances the CO_2_ transport, which results in smaller tailing factors (15%) and a decreased peak width (40%). A CGV_int_ of 60 mL min^−1^ does not further enhance the CO_2_ transport significantly but reduces the sensibility by around 12%.

##### Combined Use of Internal and External Carrier Gas

Both the internal and external carrier gas have advantages and disadvantages. Using the internal carrier gas for the transport of the generated CO_2_ from the electrolysis cell to the detector results in improved detector signals and, therefore, leads to a faster analysis. The internal carrier gas is generated finely dispersed on the electrode surfaces. For this reason, the CO_2_ gets purged effectively, highlighting the benefits of this DOC determination method. A further enhancement could be reached by an increased CGV_int_ (>60 mL min^−1^) but cannot be achieved because of the limitation through the cell voltage. Hence, a combined use of internal and external carrier gas was tested. The tests showed that 45 mL min^−1^ of each, CGV_int_ and CGV_ex_ (CGV_int + ex_ = 90 mL min^−1^), leads to even more enhanced detector signals, see Table 3. This leads to faster and more accurate analysis results and also lowers the power consumption due to the reduced analysis time.

### 3.2. Optimization of the Electrolyte Composition

**Figure 4:**
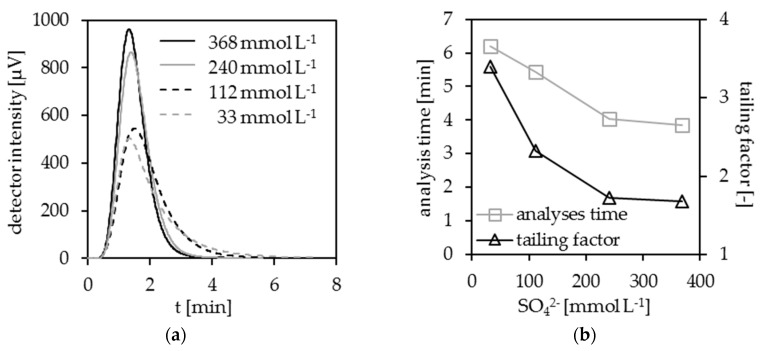
(**a**) Decomposition of pyridine as a function of the sulfate concentration; (**b**) peak parameters of dissolved organic carbon (DOC) decomposition as a function of the sulfate concentration; H_2_SO_4_, pH = 1.4, I = 4.0 A, stainless steel cathode, DiaCCon BDD.

Considering the possible generation of SO_4_^−•^ radicals, their longer lifetime, and the described decomposition mechanism, it can be assumed that the concentration of SO_4_^2−^ added to the electrolyte has a significant impact on the efficiency of the DOC decomposition. For this reason, the complete DOC decomposition in dependence on the SO_4_^2−^ concentration was investigated. The pH was kept constant at 1.4, and the sulfate concentration was varied between 33 and 368 mmol L^−1^. Figure 4 demonstrates that the sulfate concentration has a significant influence on the peak shape, the tailing factor, and the analysis time. The tailing factor decreases with increasing sulfate concentration from 3.4 to 1.7. The results for 240 and 368 mmol L^−1^ sulfate differ only slightly, whereas a significant deterioration occurs when 112 mmol L^−1^ sulfate is used instead of 240 mmol L^−1^. This is valid for the duration of the analysis, see Figure 4b. This proves the dependence of the DOC decomposition on the sulfate concentration. It also points to another, sulfate-based oxidant species, which is formed in addition to ^•^OH radicals.

**Figure 5:**
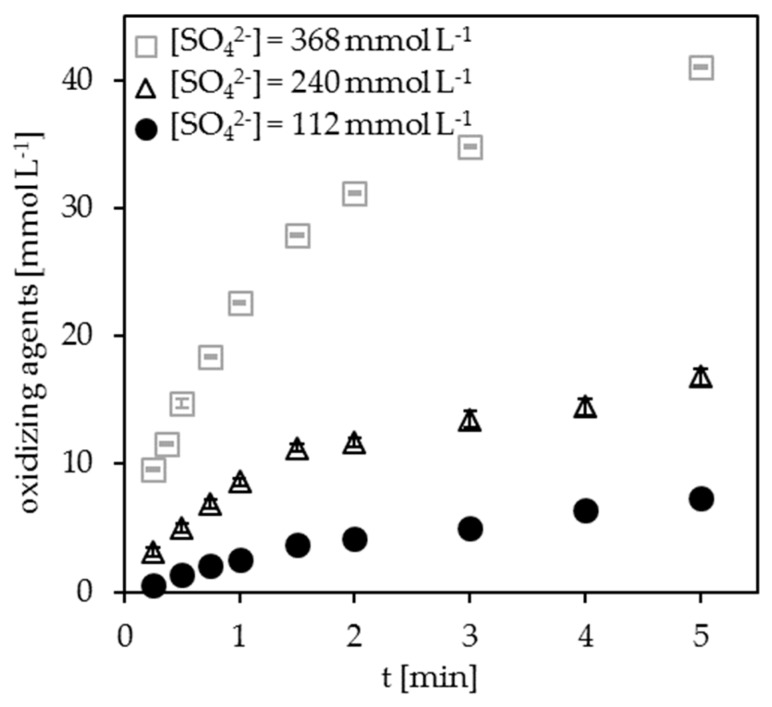
Total oxidizing agent’s concentration as a function of the electrolysis time and sulfate concentration; H_2_SO_4_, pH = 1.4, I = 4.0 A, stainless steel cathode, DiaCCon BDD.

To quantify the produced amount of secondary oxidants, which can significantly support the DOC decomposition, the total amount of measurable oxidants was analyzed. Here, only metastable oxidants, such as hydrogen peroxide or peroxodisulfate, can be detected. Short-lived substances, such as ^•^OH radicals and SO_4_^−•^ radicals, are no longer present at sampling after electrolysis at the analyzer outlet. As shown in Figure 5, the production of more stable oxidants increases with increasing concentrations of sulfate. After a certain time, the oxidizing agent concentration reaches a concentration plateau because the rate of the generation of oxidizing agents equates the rate of their cathodic decomposition. Additional measurements show that only 0.4% of the oxidizing agents is H_2_O_2_ and 99.6% is peroxodisulfate.

The more oxidizing agents are generated in the cell, the faster the DOC decomposition proceeds. Therefore, a high sulfate concentration in the electrolyte is advantageous. Interestingly, the acceleration of the decomposition kinetics shown in Figure 4a does not increase linearly with the sulfate concentration. Since the difference between the electrolytes with 368 and 240 mmol L^−1^ is rather small, the 240 mmol L^−1^ sulfate concentration represents a good compromise between sulfate concentration and decomposition efficiency. Under these optimized conditions, no significant difference in the decomposition kinetics of KHP and pyridine are detectable anymore.

### 3.3. Validation and Field Testing of the Prototype

The prototype was first calibrated and validated according to DIN 32 645 [26]. The resulting parameters are shown in Table 4. With a limit of quantification of 0.31 mg L^−1^ C and a small relative standard deviation (2.3%), the analytical method is suitable for all kinds of environmental analyses, including drinking water and sewage. In-situ UV-Vis spectrometers, which are often used for an on-site DOC determination, show a significantly higher scattering of the measurement values and are unreliable if the DOC concentration is smaller than 10 mg L^−1^ C [4]. In addition, these spectral absorbance measurements are matrix-dependent to a large degree [4]. The determination method presented in this study shows none of these disadvantages.

**Figure 6:**
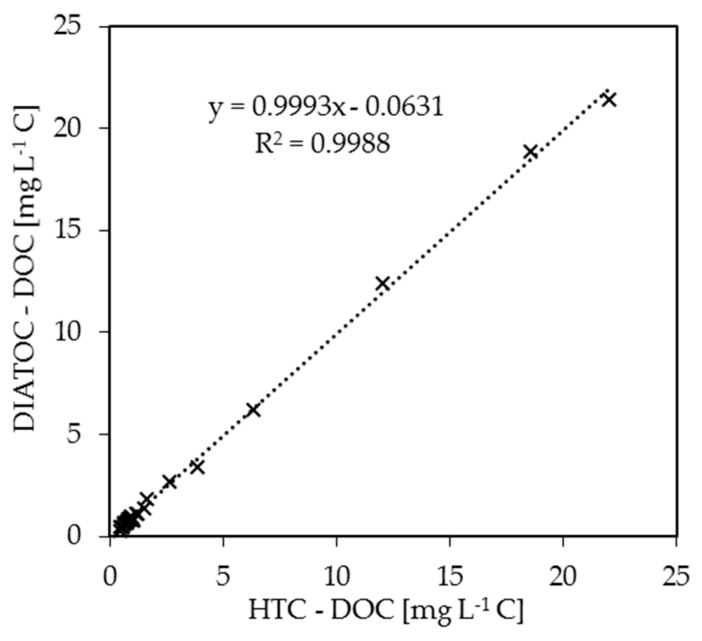
DOC monitoring data and a comparison between prototype (DIATOC) and high-temperature combustion (HTC).

The field testing was conducted in Northern India in the State of Uttarakhand at different sites along the River Ganges and some of its tributaries, see Figure 2. The on-site analysis data obtained with the prototype were compared with in-house DOC measurements using a high-temperature combustion (HTC) device. The suitability of the prototype as a DOC determination method can be observed in the correlation between the on-site analysis and the laboratory analysis, see Figure 6. It proves the general suitability of the portable prototype.

## 4. Conclusions

The aim of the present study, to create a portable prototype for on-site DOC determination with an environmentally relevant limit of detection and convenient handling, was achieved. Significantly influenced by the optimization of the electrolyte and the choice of electrode material, the prototype was tested successfully in the field. With a low relative standard deviation (2.3%), the robustness of this method was shown. The limit of quantitation (0.31 mg L^−1^ C) is appropriate for numerous applications such as surface, ground, and drinking water analysis. 

The main advantages of the validated DOC analysis method are:

- no burning technology needed
- no catalyst or ultrapure carrier gases needed
- short analysis time of under 5 min
- on-site measurement installation in under 5 min

According to the results of this study, the electrolysis cell of this device should be equipped with a BDD, which provides a surface with high crystallinity, and a stainless steel cathode for optimal analysis results and low power consumption. The novel combination of in-situ generated carrier gas and cleaned ambient air leads to a fast and reliable transport of the CO_2_ generated through the decomposition of DOC present in the injected sample. The constructed portable prototype proved its operational capability in an ongoing environmental monitoring program in Northern India. The obtained data are in agreement with those received by established methods like the high-temperature combustion. Carrying on this work, achieving automation of the sample dosing is desirable, not only to improve the user comfort but also to further enhance the accuracy of the method. 

## Figures and Tables

**Table 1:** Composition of all used electrolytes.

Electrolytes	H_2_SO_4_ 96% (μL L^−1^)	Na_2_SO_4_ (g L^−1^)	c (SO_4_^2−^) * (mmol L^−1^)	pH ** (−)	κ ** (mS cm^−1^)
pH 1.4 sulf_0_	1850	0	33	1.38	17.6
pH 1.4 sulf_1_	3150	8	112	1.40	25.8
pH 1.4 sulf_2_	3600	25	240	1.40	43.2
pH 1.4 sulf_3_	4450	41	368	1.40	43.2
pH 1.4 HNO_3_	0	0	0	1.44	17.4

* calculated; ** measured.

**Table 2:** Surface characterization of the boron-doped diamond electrode BDD-SiC-C anodes and determined reactivities; HNO_3_, pH = 1.5, κ = 17 mS cm^−1^, I = 1.0 A, stainless steel cathode.

BDD-SiC-C	REM Picture (Resolution 5 μm)	Layer Thickness (μm)	Crystallinity Grain Size (μm)	Surface Area (A_s_/A) *	k_Q_ (mL A^−1^ s^−1^)
1	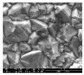	18	2.16 ± 0.28	1.219 ± 0.0009	0.029
2	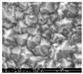	16	1.79 ± 0.33	1.159 ± 0.0012	0.028
3	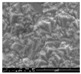	17	0.96 ± 0.16	1.049 ± 0.0004	0.022
4	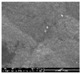	17	0.12 ± 0.05	1.019 ± 0.0005	0.017

* proportion of the calculated surface area from the determined roughness to the geometrical surface area [34].

**Table 3:** Peak parameters for different volumetric flow rates of the carrier gas.

	CGV (mL min^−1^)	Peak Height (μV)	Peak Area (μV min)	Analysis Time (min)	Tailing Factor (−)
CGV_ex_	30	168	453	9.10	3.1
	60	210	359	6.43	2.9
	90	206	233	4.27	2.8
	120	200	199	4.03	3.2
	150	182	167	3.88	3.3
CGV_int_	30	217	439	6.30	1.4
	45	237	393	3.97	1.2
	60	233	345	3.55	1.2
CGV_int + ex_	90 (45 + 45)	252	222	3.05	1.2

**Table 4:** Parameters of the validation according to DIN 32 645 [26].

Parameter	Value	Unit
Correlation coefficient	0.9992	-
Relative standard deviation	2.3	%
Limit of detection	0.10	mg L^−1^ C
Limit of quantification	0.31	mg L^−1^ C

## References

[1] Visco G., Campanella L., Nobili V. (2005). Organic carbons and TOC in waters: An overview of the international norm for its measurements. Microchem. J..

[2] Lin H., Wu J., Zhang H. (2013). Degradation of bisphenol A in aqueous solution by a novel electro/Fe^3+^/peroxydisulfate process. Sep. Purif. Technol..

[3] Urbansky E.T. (2001). Total organic carbon analyzers as tools for measuring carbonaceous matter in natural waters. J. Environ. Monit..

[4] Avagyan A., Runkle B.R.K., Kutzbach L. (2014). Application of high-resolution spectral absorbance measurements to determine dissolved organic carbon concentration in remote areas. J. Hydrol..

[5] Vogl J., Heumann K.G. (1998). Development of an ICP−IDMS method for dissolved organic carbon determinations and its application to chromatographic fractions of heavy metal complexes with humic substances. Anal. Chem..

[6] Tepuš B., Simonič M. (2007). Uncertainty of the result of TOC determination in water samples. Accredit. Qual. Assur..

[7] Schmalz V., Börnick H., Worch E. (2008). Technische Universität Dresden. Verfahren und Vorrichtung zur Bestimmung des Gesamten Organisch Gebundenen Kohlenstoffs in Einer Wässrigen Probe. Patent.

[8] Comninellis C. (1994). Electrocatalysis in the electrochemical conversion/combustion of organic pollutants for waste water treatment. Electrochim. Acta.

[9] Chen X., Chen G., Gao F., Yue P.L. (2003). High-performance Ti/BDD electrodes for pollutant oxidation. Environ. Sci. Technol..

[10] Boye B., Brillas E., Marselli B., Michaud P.-A., Comninellis C., Farnia G., Sandonà G. (2006). Electrochemical incineration of chloromethylphenoxy herbicides in acid medium by anodic oxidation with boron-doped diamond electrode. Electrochim. Acta.

[11] Iniesta J., Michaud P.A., Panizza M., Comninellis C. (2001). Electrochemical oxidation of 3-methylpyridine at boron-doped diamond electrode: Application to electroorganic synthesis and wastewater treatment. Electrochem. Comm..

[12] Panizza M., Michaud P.A., Cerisola G., Comninellis C. (2001). Anodic oxidation of 2-naphthol at boron-doped diamond electrodes. J. Electroanal. Chem..

[13] Guinea E., Arias C., Cabot P.L., Garrido J.A., Rodríguez R.M., Centellas F., Brillas E. (2008). Mineralization of salicylic acid in acidic aqueous medium by electrochemical advanced oxidation processes using platinum and boron-doped diamond as anode and cathodically generated hydrogen peroxide. Water Res..

[14] El-Ghenymy A., Garrido J.A., Centellas F., Arias C., Cabot P.L., Rodríguez R.M., Brillas E. (2012). Electro-Fenton and photoelectro-Fenton degradation of sulfanilic acid using a boron-doped diamond anode and an air diffusion cathode. J. Phys. Chem. A.

[15] Glorian H., Schmalz V., Kürbis S., Börnick H., Worch E., Dittmar T. (2017). Electrochemical decomposition of dissolved organic carbon using boron-doped diamond technology as basic element of a portable DOC analyzer. J. Electroanal. Chem..

[16] DIN Deutsches Institut für Normung, e.V., Arbeitsausschuss Chemische Terminologie(AChT) D (1997). I. für Normung, DIN EN 1484-1997: Anleitungen zur Bestimmung des Gesamten Organischen Kohlenstoffs (TOC) und des Gelösten Organischen Kohlenstoffs (DOC).

[17] Lipczynska-Kochany E., Sprah G., Harms S. (1995). Influence of some groundwater and surface waters constituents on the degradation of 4-chlorophenol by the Fenton reaction. Chemosphere.

[18] Kerwick M.I., Reddy S.M., Chamberlain A.H.L., Holt D.M. (2005). Electrochemical disinfection, an environmentally acceptable method of drinking water disinfection?. Electrochim. Acta.

[19] Michaud P.-A., Mahé E., Haenni W., Perret A., Comninellis C. (2000). Preparation of peroxodisulfuric acid using boron-doped diamond thin film electrodes. Electrochem. Solid-State Lett..

[20] Palmas S., Polcaro A.M., Vacca A., Mascia M., Ferrara F. (2007). Influence of the operating conditions on the electrochemical disinfection process of natural waters at BDD electrodes. J. Appl. Electrochem..

[21] Davis J., Baygents J.C., Farrell J. (2014). Understanding persulfate production at boron doped diamond film anodes. Electrochim. Acta.

[22] Neta P., Huie R.E., Ross A.B. (1988). Rate constants for reactions of inorganic radicals in aqueous solution. J. Phys. Chem. Ref. Data.

[23] Mascia M., Vacca A., Palmas S., Polcaro A.M. (2006). Kinetics of the electrochemical oxidation of organic compounds at BDD anodes: Modelling of surface reactions. J. Appl. Electrochem..

[24] Lutze H. (2013). Sulfate Radical Based Oxidation in Water Treatment. Ph.D. Thesis.

[25] Serrano K., Michaud P.A., Comninellis C., Savall A. (2002). Electrochemical preparation of peroxodisulfuric acid using boron doped diamond thin film electrodes. Electrochim. Acta.

[26] DIN Deutsches Institut für Normung, e.V., Arbeitsausschuss Chemische Terminologie(AChT) (1994). DIN 32645 1994-05: Chemische Analytik; Nachweis-, Erfassungs- und Bestimmungsgrenze; Ermittlung unter Wiederholbedingungen, Begriffe, Verfahren, Auswertung.

[27] Liang C., Huang C.-F., Mohanty N., Kurakalva R.M. (2008). A rapid spectrophotometric determination of persulfate anion in ISCO. Chemosphere.

[28] Bennedsen L.R., Søgaard E.G., Muff J. (2014). Development of a spectrophotometric method for on-site analysis of peroxygens during in-situ chemical oxidation applications. Water Sci. Technol..

[29] Klassen N.V., Marchington D., McGowan H.C.E. (1994). H_2_O_2_ Determination by the I_3_-Method and by KMnO_4_ Titration. Anal. Chem..

[30] Sellers R.M. (1980). Spectrophotometric determination of hydrogen peroxide using potassium titanium(IV) oxalate. Analyst.

[31] Brandhuber P., Korshin G.V. (2009). Methods for the Detection of Residual Concentations of Hydrogen Peroxide in Advanced Oxidation Processes.

[32] Fryda M., Schäfer L., Herrmann D., Gandini D., Perret A., Comninellis C., Klages C.-P., Heanni W. (1999). Properties of diamond electrodes for wastewater treatment. New Diam. Front. Carbon Technol..

[33] Lochyński P., Dittmar T., Worch E., Kuczewski K. (2015). Electrochemical oxidation of organic compounds on boron-doped diamond electrodes. Przem. Chem..

[34] DIN Deutsches Institut für Normung e.V., Arbeitsausschuss Chemische Terminologie(AChT) (2012). EN ISO 25178-2:2012: Geometrische Produktspezifikation (GPS)—Berflächenbeschaffenheit: Flächenhaft—Teil 2: Begriffe und Oberflächen-Kenngrößen.

